# Homogeneous Catalysis and Heterogeneous Recycling: A Simple Zn(II) Catalyst for Green Fatty Acid Esterification

**DOI:** 10.1021/acssuschemeng.1c01140

**Published:** 2021-04-22

**Authors:** Massimo Melchiorre, Maria Elena Cucciolito, Martino Di Serio, Francesco Ruffo, Oreste Tarallo, Marco Trifuoggi, Roberto Esposito

**Affiliations:** † ISUSCHEM S.r.l., Piazza Carità 32, 80134 Napoli, Italy; ‡ Dipartimento di Scienze Chimiche, Università di Napoli Federico II, Via Cintia 21, 80126 Napoli, Italy; § Consorzio Interuniversitario di Reattività Chimica e Catalisi, Via Celso Ulpiani 27, 70126 Bari, Italy

**Keywords:** zinc(II), fatty acids, esterification, catalysis, catalyst recycle

## Abstract

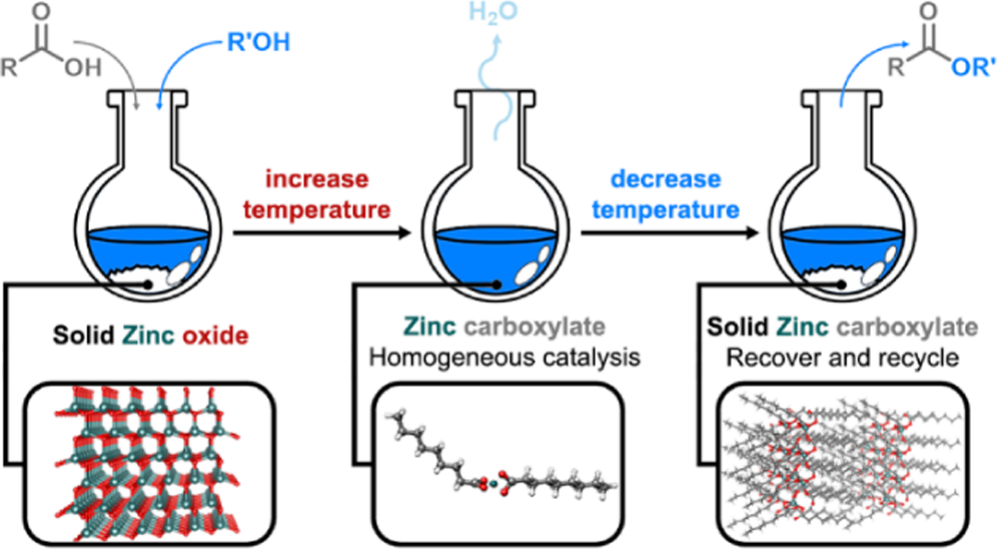

This work describes the use of simple zinc(II) salts (ZnCl_2_, ZnCO_3_, Zn(OAc)_2_, ZnO, Zn(ClO_4_)_2_, Zn(TfO)_2_, and Zn(BF_4_)_2_) as effective catalysts for the esterification of fatty acids with long-chain alcohols and simple polyols through a homogeneous system that allows the gradual and selective removal of water. The results show that the catalytic activity depends on the nature of the counterion: the most effective are the salts with poorly coordinating anions (perchlorate and triflate) or containing basic Brønsted anions (oxide, acetate, and carbonate). However, only with the latter is it possible to fully recover the catalyst at the end of each run, which is easily filtered in the form of zinc carboxylate, given its insolubility in the ester produced. In this way, it is possible to recycle the catalyst numerous times, without any loss of activity. This beneficial prerogative couples the efficiency of the homogeneous catalysis with the advantage of the heterogeneous catalysis. The process is, therefore, truly sustainable, given its high efficiency, low energy consumption, ease of purification, and the absence of auxiliary substances and byproducts.

## Abstract 2

Simple zinc(II) salts are effective and recyclable catalysts for the solvent-free esterification of fatty acids with medium−long-chain alcohols.

## Introduction

The growing concern about global warming and fossil resource consumption has led to the development of sustainable technologies, processes, and products that exploit renewable energies and feedstocks.^1,2^

Among renewable resources, fatty acids (FAs) from vegetable oils occupy a relevant position due to their similarity to the aliphatic fractions of fossil materials.^3,4^ For a full application of the principles of the circular economy, it is necessary that the vegetable raw material comes from waste or nonfood oils, a condition that creates a virtuous route for the sustainable production of many commodities.^5^ In fact, fatty acids are chemically versatile, thanks to the presence of the carboxylic group, suitable for esterification^6^ and transesterification^6^ reactions to obtain biodiesel^7−9^ and other esters for industrial applications.^10^ The presence of one or more unsaturation is instead generally exploited in oxidative processes, such as epoxidation,^11^ dihydroxylation,^12^ and C=C cleavage.^13−18^

**Scheme 1:**
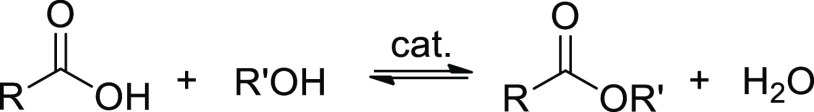
Esterification Reaction

Among the several categories of products achievable from fatty acids, esters with medium–long-chain alcohols or polyols already cover many roles in the industry, such as lubricants, solvents, surfactants, additives, and more. Their main synthetic route is the direct esterification of fatty acids (Scheme 1). This is a convenient reaction with generally high atom economy since the only byproduct is water. It is an equilibrium reaction, and therefore it is necessary to remove water from the reaction mixture to increase the yield. Moreover, an acid catalyst is often necessary to complete the reaction in acceptable time.

The literature offers numerous insights into the application of acid catalysts for the direct synthesis of these esters, which can benefit from homogeneous or heterogeneous systems, based on both Lewis and Brønsted acids, including ionic liquids. Table 1 shows some examples of catalyzed esterification of medium-/long-chain-length fatty acid with C4–C16 alcohol.

**Table 1:**
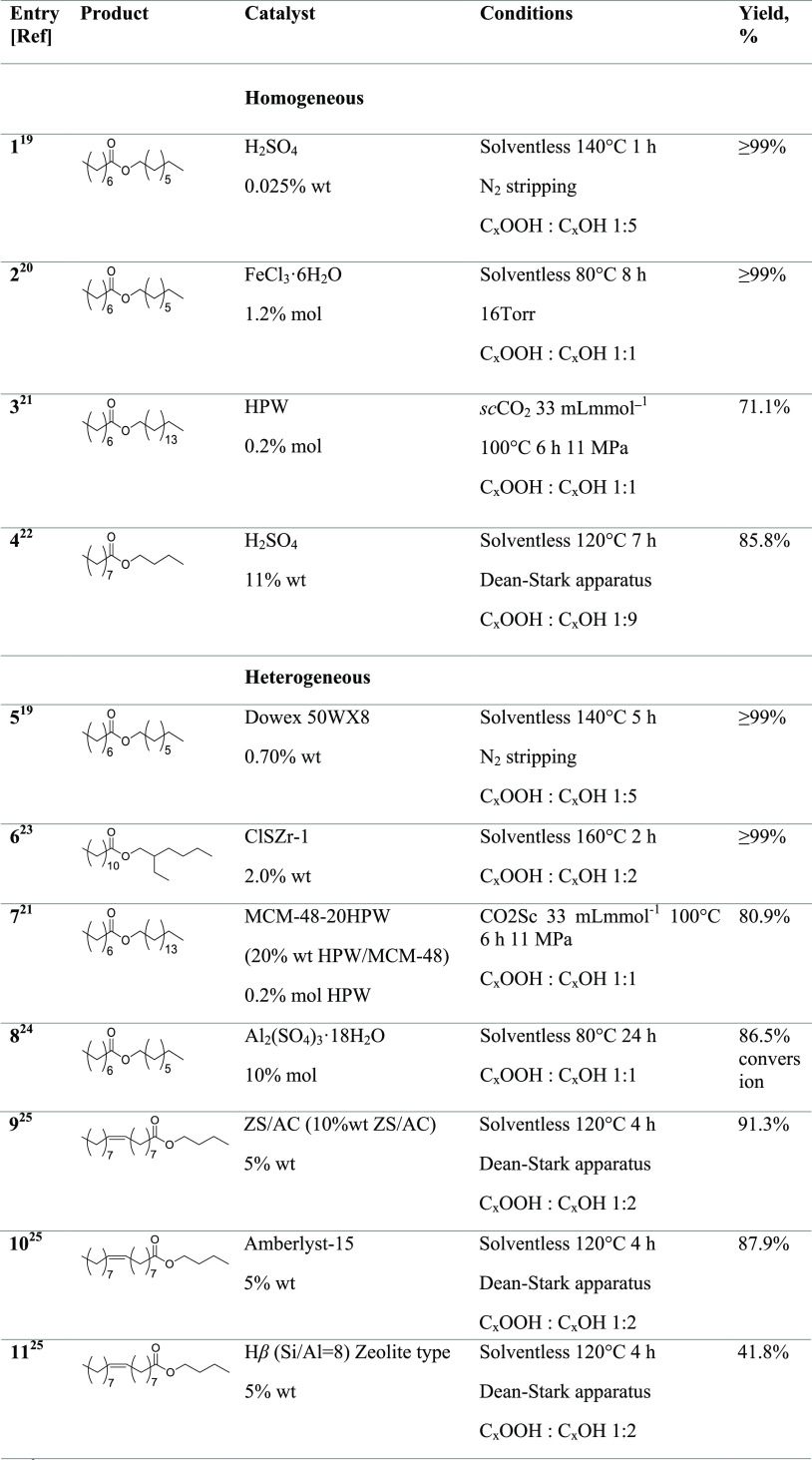

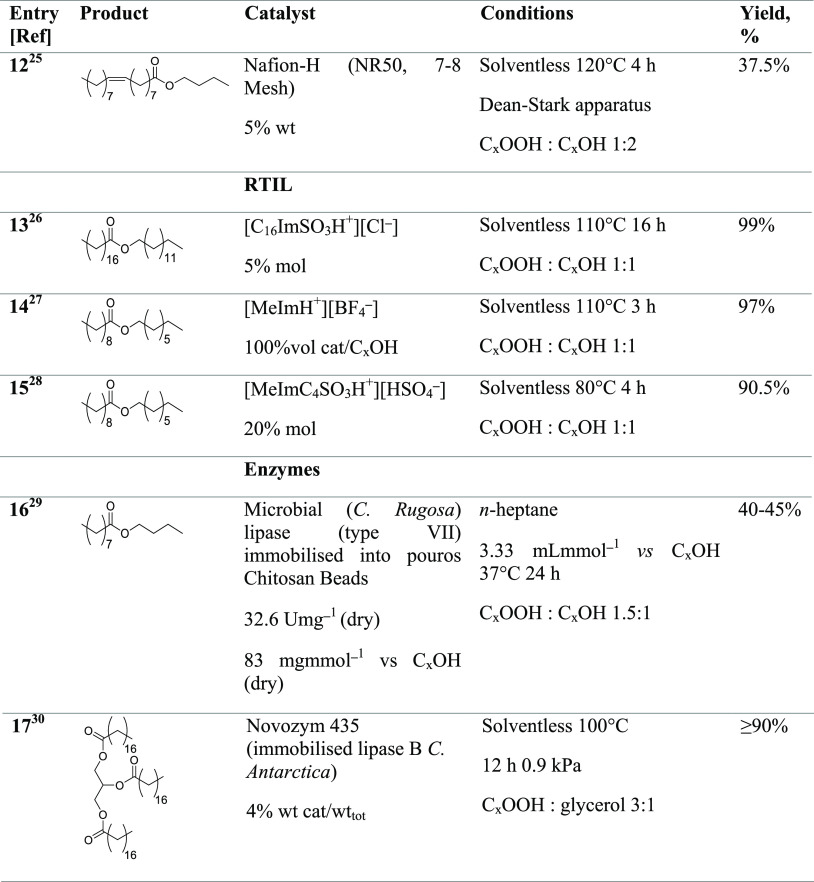
Examples of Catalyzed Esterification of Medium-/Long-Chain-Length Fatty Acid with C4–C16 Alcohol^19−30^

Each category of catalysts presents drawbacks. Despite good activity, homogeneous acids suffer various problems related to the tedious work-up procedure of purification, their corrosive action, and risks connected to their manipulation. On the contrary, heterogeneous acid catalysts are easily removed from the reaction mixture and can be recycled several times. Nevertheless, they are often subjected to leaching and poisoning phenomena that require reactivation procedures.^31^

The use of acid ionic liquids can simplify separation and recycling of the catalyst. This benefit accompanies high loading and cost that make the application unaffordable on a large scale.

Supported lipases are particularly effective in glyceride synthesis in terms of both conversions and recyclability (Bi reports a relative activity of 74% for the 14th recycling in the synthesis of the tri-acyl-stearate without washing procedures). In this case, the high cost and catalyst loading are mitigated by the possibility of numerous recycles (over 10) with simple methods. However, screening with simple alkyl alcohols is rarely reported and, when reported, satisfactory conversions are not achieved.

Within this frame, our research team is active in the valorization of vegetable oils through the conversion of fatty acids^32−34^ and glycerol,^35,36^ promoted by Lewis acid catalysis. The focus of the activity is the search for catalytic systems allowing the large-scale extension of processes^37,38^ for the synthesis of widely used products.^39^

**Scheme 2:**
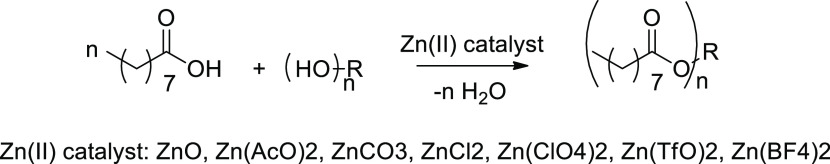
Esterification Catalyzed by Zn(II) Salts

A recent survey of the literature^6^ made it possible to identify the Zn(II) ion as a high-performing catalyst for the esterification of fatty acids with medium-/long-chain alcohols or polyols.^24,40−45^ This is due to its intermediate acidity, wide availability, and economic convenience. However, there are no studies that simultaneously address the fate of the zinc(II) catalyst, the possibility of its recycling, the contamination of the isolated product, and the effects of the Zn(II) counterion on these aspects that are essential for a possible industrial application. In this work, we fill this gap through a systematic study using pelargonic acid as a benchmark in combination with medium–long-chain alcohols and polyols (Scheme 2).

The catalytic activity of several Zn(II) salts has been investigated and their performance has been rationalized according to the nature of the counterion. The most convenient were revealed to be carbonate, acetate, and oxide, and this was justified considering their basic Brønsted properties: at the end of the reaction, the precipitation of the corresponding zinc(II) carboxylate offered the possibility to recover and reuse the solid several runs with the retention of the activity, a condition that represents a solid premise for the extension of the system on a large scale according to the principles of green chemistry.^46^

## Experimental Section

### General

Reagents and solvents were purchased from Sigma-Aldrich. The heating band was purchased from Watlow and controlled through a coupled thermocouple and a thermostat. All compounds were characterized by NMR with a Bruker Avance Ultrashield 400 operating at a proton frequency of 400 MHz or with a Varian 500 Oxford at a proton frequency of 500 MHz.

Single-beam spectra were recorded on a Nicolet Avatar 360 FT-IR spectrometer in a range of 4000–400 cm^–1^, with a 2 cm^–1^ resolution. Samples were prepared using the Nujol method and KBr plates.

The residual water content in the filtered crude reaction mixtures was evaluated through Karl-Fischer titration with a Metrohm 831KF Coulometer. The absolute water content of each sample was comprised between 30 and 100 μg.

Residual concentrations of zinc in the products were determined by a set of inductively coupled plasma mass spectrometry (ICP-MS) analyses using an Aurora M90 Brucker apparatus.

Samples were subjected to oxidative acid digestion with a mixture of 69% nitric acid and 30% v/v hydrogen peroxide in a 8:1 ratio, using high temperature and pressure, under a microwave-assisted process. A proper dilution was made and the suspension obtained for each sample was introduced to the plasma. The quantitative analysis was performed using an external calibration curve using multielement standard solutions for ICP TraceCERT in 5% nitric acid (Sigma-Aldrich, Milan, Italy) and ultrapure deionized water with conductivity <0.06 μS/cm.

### Catalytic Runs and Analysis

**Figure 1:**
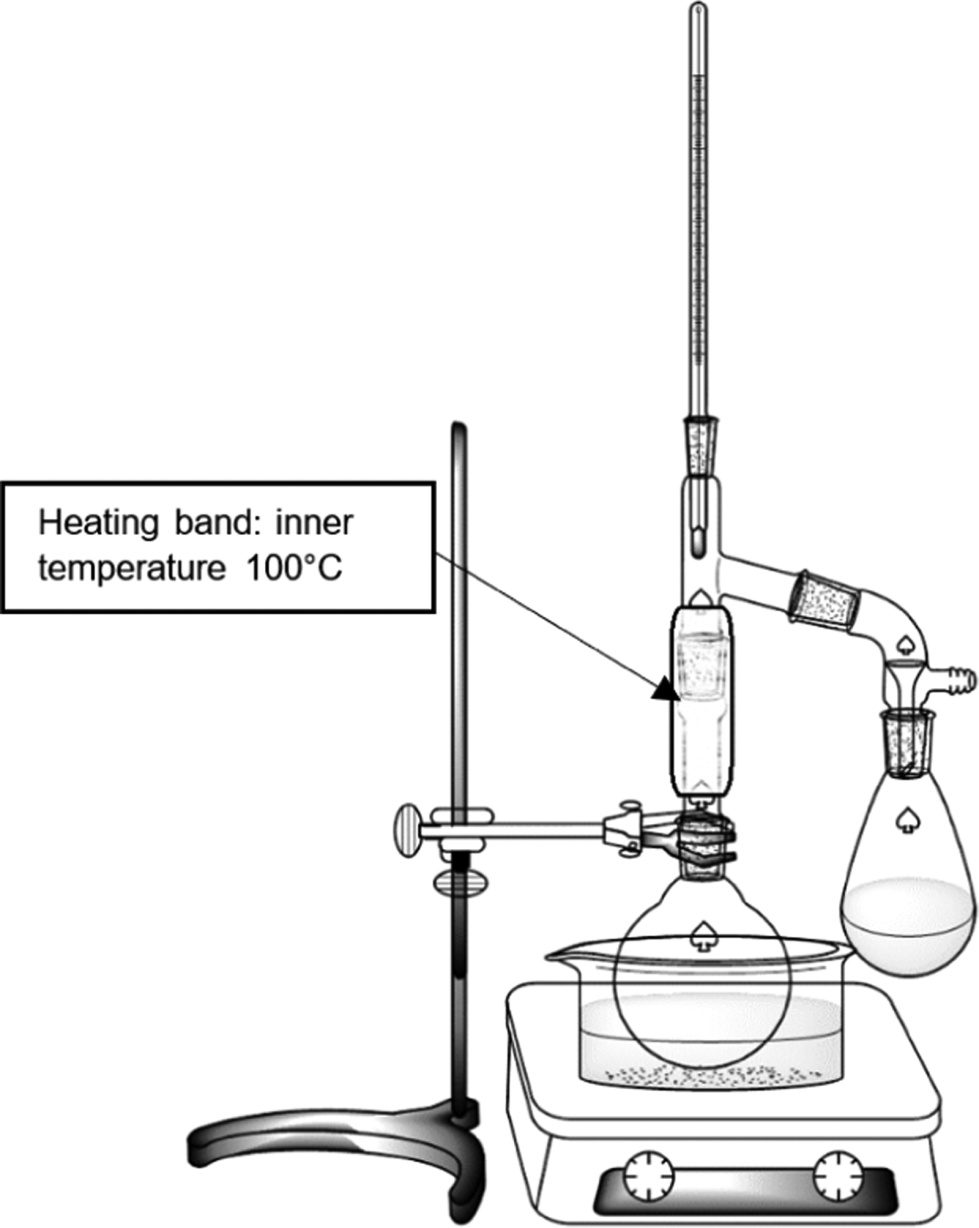
Reaction apparatus.

All reactions were performed in a 100 mL round-bottom flask and the quantity in moles of fatty acid and alcohol (*n*_FA_, *n*_ROH_) was calculated to reach a total volume of 50 mL, considering the reagents’ densities (ρ_FA_, ρ_ROH_), their molecular weights (mw_FA_, mw_ROH_), and the used molar ratio (MR). This setup was done to keep the reaction apparatus unaltered while changing different fatty acids and alcohols. The used equations are reported below (eqs 1 and 2)
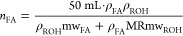
1
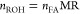
2Appropriate amounts of fatty acid and alcohol were added to the reaction flask and premixed for few minutes. Next, an appropriate amount of a catalyst was added to the reaction mixture and a still head equipped with a heating band was fitted on the flask. Downstream of the still head, a graduated 5 mL Schlenk tube was mounted to monitor the conversion by collecting the water produced by the reaction (Figure 1). The reaction time was considered after 15 min from the moment that the reaction mixture was placed in the preheated oil bath, and the mixture was left to react at the set temperatures for the appropriate time. This 15 min delay was chosen to allow the achievement of the thermal equilibrium between the temperature of the hot bath and the temperature of the reaction mixture.

The reactions were performed at 170 °C and the heating band was set to keep the still head inner temperature at 100 °C, allowing the water distillation and the alcohol condensation.

The conversion is verified by taking a sample from the reaction mixture at different times and analyzing it through ^1^H NMR spectroscopy. At the end of the reaction time, the reaction mixture is allowed to cool overnight to room temperature and filtered under vacuum with P3 sintered glass filters. The zinc concentration in the filtered phase was evaluated by ICP-MS analysis. The resulting filtered spent catalyst was washed, dried, and analyzed with XRD and FT-IR to confirm the Zn(II) compound nature.

The isolated yield of 2-ethylhexyl pelargonate was achieved through vacuum distillation of excess alcohol and catalyst filtration. At the end of reaction time and without lowering the temperature, the pressure was gently lowered to 100 mbar and held for 15 min, successively lowered to 50 mbar and held for 30 min, and finally lowered to 30 mbar and held for 5 min. After this procedure, the system was allowed to cool at room temperature and filtered to recover the catalyst.

As an example of a catalytic run to synthesize 2-ethylhexyl pelargonate with FA/alcohol MR of 1.2 and ZnO as a catalyst, 125 mmol pelargonic acid (19.8 g, 22.0 mL), 150 mmol 2-ethylhexyl alcohol (19.5 g; 23.5 mL), and 1.25 mmol ZnO (0.102 g) were added into a 100 mL round-bottom flask.

### Spent Catalyst Analysis

Before each analysis, the spent catalysts were washed with ethyl acetate, dried under vacuum, and finally in an oven overnight at 50 °C.

^1^H NMR analysis: The spent catalyst (10 mg) was suspended in CDCl_3_ (1 mL) and treated with a concentrated solution of hydrochloric acid until complete solid dissolution. The organic layer was recovered and dried with anhydrous sodium sulfate before analysis.

Wide-angle X-ray diffraction patterns of catalysts (as synthesized and recovered after reactions) were obtained with an automatic Philips powder diffractometer (Philips PW1830) operating in the θ/2θ Bragg–Brentano geometry using nickel-filtered Cu Kα radiation and performing a continuous scan in the 2θ range 1.6–50°. Specimen holders were 2 mm thick. When crystals were in the form of small plates, before being analyzed, samples were finely ground.

### Catalyst Recovery and Recycle

To investigate the feasibility of recycling the catalyst, a set of five consecutive runs were performed in optimized conditions and with the reaction apparatus previously described. For each run, 70 mmol pelargonic acid (11.1 g; 12.2 mL) and 88 mmol 2-ethylhexyl alcohol (10.9 g; 13.1 mL) were placed in a 50 mL round-bottom flask. Only for the first run (run 0), 0.70 mmol ZnO (57 mg) was used as a fresh catalyst. At the end of each run, the reaction mixture was allowed to cool overnight at room temperature; the resulting precipitated zinc(II) carboxylate was filtered, washed with ethyl acetate and *n*-hexane, dried under vacuum, weighed, and reintroduced quantitatively into the flask as a catalyst for the next run.

## Results and Discussion

### Catalyst Screening

A first catalytic screening was performed on a series of Zn(II) salts to evaluate the effect of the counterion on both activity and leaching. The comparison was carried out considering coordinating properties and Brønsted basicity of the anion, which allowed identifying the best catalyst for subsequent optimizations.

**Scheme 3:**
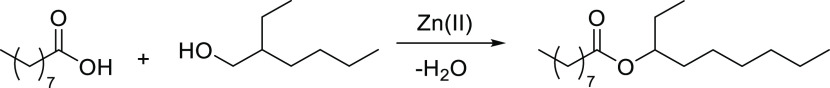
Esterification of Pelargonic Acid with 2-Ethylhexyl Alcohol

Esterification reactions were conducted using pelargonic acid (PA) and 2-ethylhexyl alcohol as benchmarks for fatty acids and alcohols (Scheme 3). An excess of 20% mol alcohol was used.

The reactions were settled up to allow the water distillation and the alcohol condensation (with bp >120 °C). The temperature was chosen as a compromise: as high as possible to favor the kinetics, but adequately lower than the boiling point of the alcohol (187 °C) to avoid excessive evaporation.

Under the applied conditions, the Brønsted acid contribution is considerable. In the absence of a Zn(II) catalyst, a yield of 68% is achieved in 2 h. However, this contribution decreases over time due to the progressive consumption of carboxylic acid and its dilution in the ester produced: after 4 h the yield does not exceed 84%.

Instead, the yield is substantially higher within 4 h with a Zn(II) loading of 1% mol. Table 2 shows both yields and leaching of the catalyst in the isolated product.

**Table 2:** Catalyst Screening

		yield (%)^b^		
entry	catalyst^a^	2 h	4 h	[Zn] in product (ppm)^c^
1		68	84	
2	ZnO	80	94	1.4
3	Zn(AcO)_2_	78	95	2.5
4	ZnCO_3_	75	94	7.6
5	ZnCl_2_	73	88	soluble^d^
6	Zn(BF_4_)_2_	73	85	0.3
7	Zn(ClO_4_)_2_	99	>99	soluble^d^
8	Zn(CF_3_SO_3_)_2_	>99	>99	soluble^d^

a Conditions: 170 °C, fatty acid to alcohol 1:1.2 mol/mol, and catalyst loading 1% mol.

b Thorough ^1^H NMR spectroscopy, relative error within 2%.

c ICP-MS analysis.

d Not evaluated because the catalyst was completely soluble in the cold mixture.

**Figure 2:**
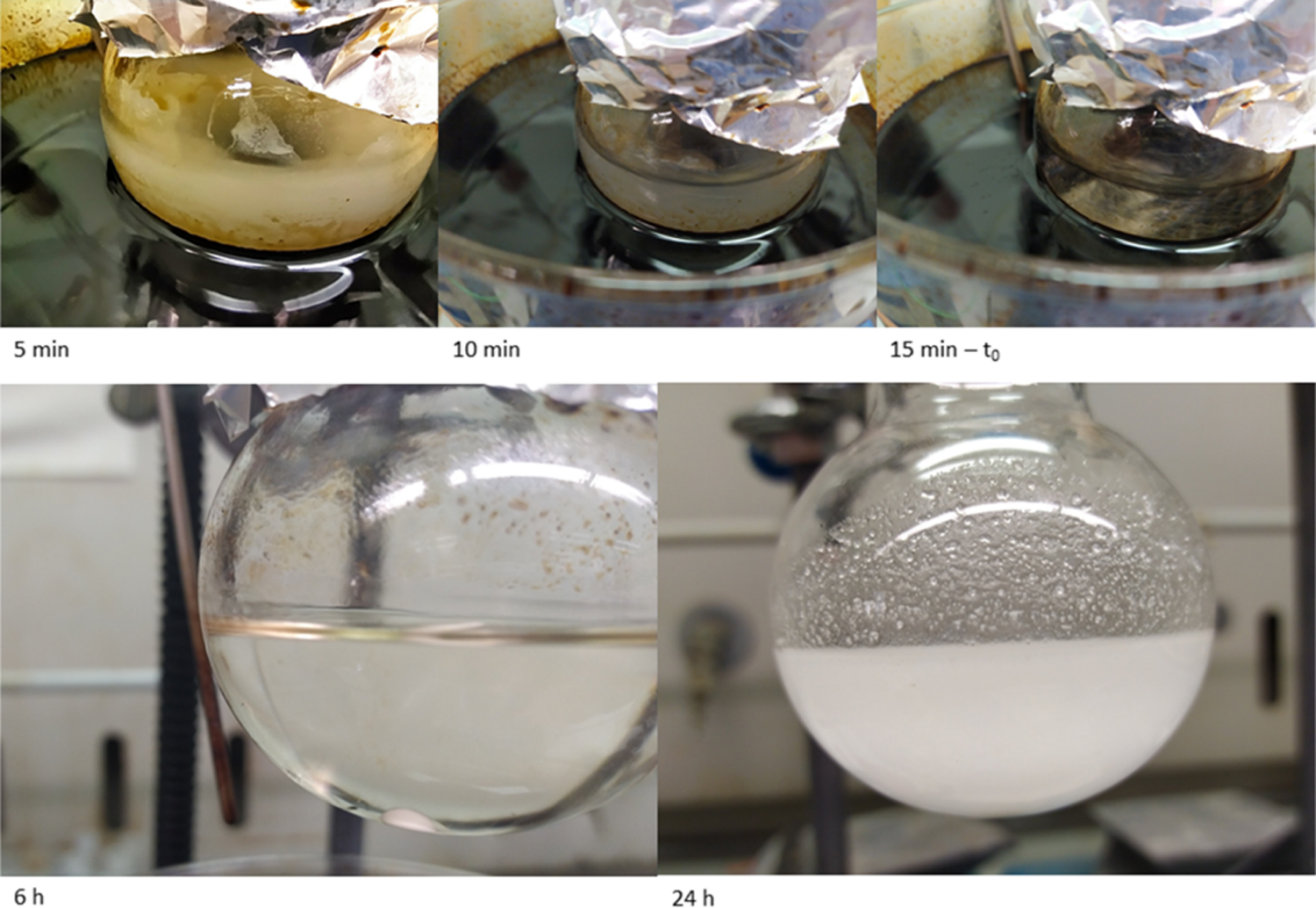
Complete dissolution of the initial catalyst within 15 min at 165–170 °C and then precipitation at room temperature of the filtrable white solid (zinc carboxylate).

Yields >94%, within 4 h, were obtained with salts containing Brønsted basic counterions, such as zinc oxide, acetate, and carbonate. A change in the appearance of the reaction mixture was noted after about 15 min at 170 °C, passing from an opalescent dispersion of the solid catalyst to its complete dissolution. Upon cooling the reaction mixture, a white crystalline solid could be easily filtered, whose mass was found higher than that of the salt initially used (Figure 2).

**Scheme 4:**
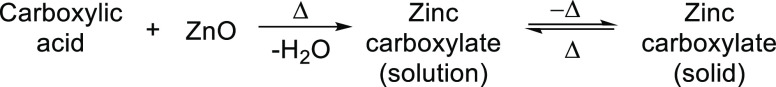
Zinc Carboxylate Formation

^1^H NMR spectra (Figure S1) and XRD analysis of the solids (Figure S2) disclosed their structure as Zn(pelargonate)_2_, formed *in situ* during the reaction (Scheme 4). Indeed, similarly to what was observed for saturated fatty acid zinc salts,^47,48^ Zn(pelargonate)_2_ showed a distinctive diffraction pattern, typical of the layered crystalline structure, with an interlayer Bragg distance equal to 2.24 nm, as revealed by the reflection at 2θ = 3.95°. These values nicely fit with the number of carbon atoms according to the dependence reported in the literature.^48^ The reactivity of zinc salts with a basic anion is shown in the literature^49−51^ but not yet reported for the used acid.

Zinc perchlorate and zinc triflate produce high yields as well, probably due to the high “nudity” of the Zn(II) ion, given the poor coordinating ability of the perchlorate and triflate ions. Their concurrent low Brønsted basicity does not allow the *in situ* formation of zinc pelargonate, and the reaction mixture behaves very differently: a gradual darkening accompanies the progress of the reaction, and, in the end, precipitation of any solid is not observed. Apparently conflicting is the result obtained with tetrafluoroborate, which is comparably weak both as Lewis and Brønsted base: tetrafluoroborate promotes a yield similar to that obtained in the absence of a catalyst. This discrepancy can be due to its scarce solubility in the reaction conditions.

Intermediate results were obtained with zinc chloride, which is more soluble than tetrafluoroborate but displays a more coordinating anion than perchlorate. Again, no zinc pelargonate formation was observed, probably due to the weakness of chloride as a Brønsted base.

Although higher activities were observed for perchlorate and triflate salts, their complete solubility in the reaction media prevents the possibility to recover and reuse these catalysts.

Since leaching was negligible in the case of zinc oxide (Table 2), it was selected as the suitable candidate for continuing the study. Furthermore, zinc oxide presents other benefits of low cost, large availability, and a good ecotoxicological profile.

### Optimization of the Catalytic Conditions

The reaction conditions were optimized with respect to both the quantity of the catalyst and the acid/alcohol molar ratio. The catalyst loading was varied in comparative runs using 0.1, 1.0, and 2.5% mol catalyst with respect to the acid, keeping an acid/alcohol molar ratio of 1:1.2. The yields obtained at 2 and 4 h are reported in Table 3.

**Table 3:** Zinc Oxide Screening

		yield (%)^b^	
entry^a^	ZnO (%)	2 h	4 h
1		68	84
2	0.1	71	89
3	1.0	80	94
4	2.5	94	98

a Condition: 170 °C, fatty acid to alcohol 1:1.2 mol/mol.

b Thorough ^1^H NMR spectroscopy, relative error within 2%.

In each case, Zn(II) pelargonate was easily filtered at the end of the reaction; even with 2.5% mol catalyst, the crude mixture was filtered in short time. However, 1% mol loading was considered as the right compromise to have a reasonable reaction rate and catalyst loading and to prevent excessive consumption of fatty acid in the formation of the Zn(II) carboxylate species.

**Figure 3:**
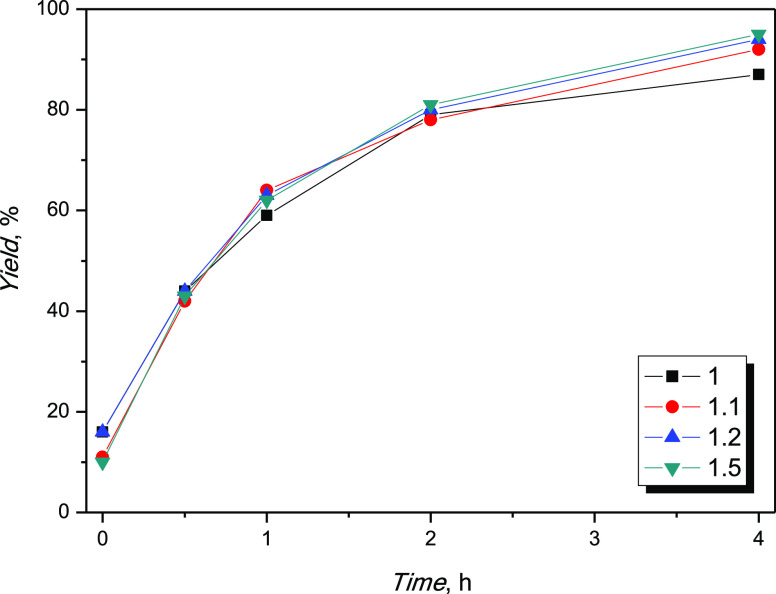
Alcohol/acid molar ratio screening.

The effect of the acid/alcohol molar ratio was evaluated ranging from stoichiometric amounts up to 1:1.5. An excess of 10% mol significantly increases the yield and an excess of 20% mol was already enough to achieve high yields in 4 h (Figure 3). Further increasing the acid/alcohol molar ratio does not have an appreciable beneficial effect. Based on these findings, an excess of 20% mol alcohol was chosen as the optimal condition to assess the scope of the method.

Under the best conditions, it is possible to reach a complete conversion within 6 h of the reaction (Figure S3). The product was isolated almost quantitively upon vacuum distillation of excess alcohol and catalyst filtration at room temperature.

As shown in Figure S3, it is also possible to appreciate the prominent contribution of Zn(II) Lewis acid catalysis by comparing the experiment performed without the catalyst.

### Scope of Fatty Acids and Alcohols

The esterification of a panel of acids and alcohols was performed under the optimized conditions using zinc oxide as a catalyst. Pelargonic acid was fixed in the screening of alcohols while 2-ethylhexyl alcohol was fixed for acids.

**Figure 4:**
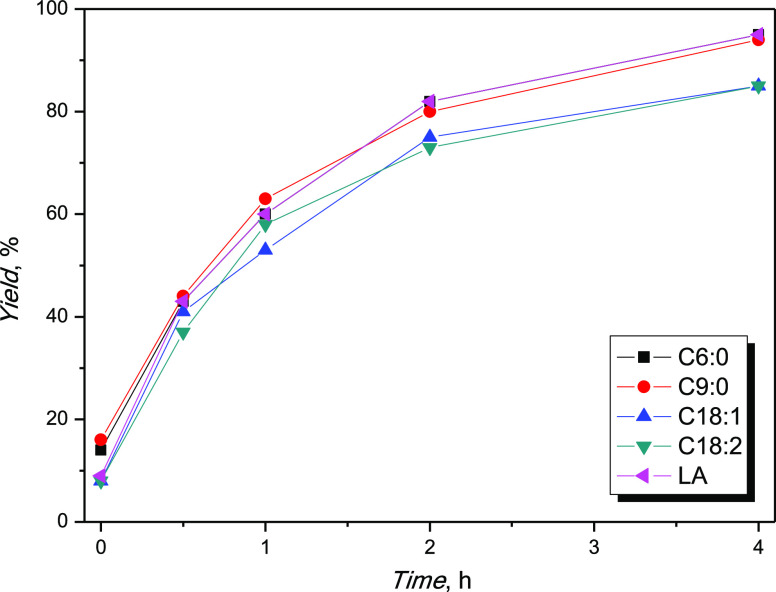
Fatty acid screening. C4:0 yield over time is not reported due to high vapor pressure of butyric acid.

Butyric (C4:0), caproic (C6:0), pelargonic (C9:0), oleic (C18:1), linoleic (C18:2), and levulinic acid (LA) were selected on the basis of their chain lengths, the presence of unsaturation and, in the case of levulinic acid, due to its relevance as a functionalized derivative of lignocellulosic biomass. Table 4 shows the yields after 2 and 4 h, while Figure 4 shows the yield trends over time.

**Table 4:** Scope of Fatty Acids

		yield (%)^b^		
entry^a^	fatty acid	2 h	4 h	[Zn] in product (ppm)^c^
1	C4:0		90	73.8
2	C6:0	82	95	19.7
3	C9:0	80	94	1.4
4	C18:1	75	85	8.4
5	C18:2	73	85	8.4
6	LA	88	97	4.8

a Condition: 170 °C, fatty acid to alcohol 1:1.2 mol/mol, and catalyst loading ZnO 1% mol.

b Thorough ^1^H NMR spectroscopy, relative error within 2%.

c ICP-MS analysis.

Pleasingly, the catalytic system was found to be compatible with double bonds (C18:1), allylic hydrogen atoms (C18:2), and carbonyl groups (LA). High yields were obtained with the entire set of carboxylic acids, although a slight decrease in the performance is appreciable for the shorter chain (C4:0) and the longer ones (C18:1 and C18:2). This trend probably reflects the lower boiling point of butyric acid and presumably reduced accessibility to the catalyst in the presence of the long and flexible chains, admitting that also, in these cases, the effective catalysts are the corresponding zinc(II) carboxylates produced *in situ*. In fact, the FT-IR spectra of the recovered catalyst were in accordance with literature^52−54^ data (Figure S4).

The water content in the products (Table 4—entries 1–6) was verified. As expected, the water content was small in all cases (0.28–0.04% wt), slightly increasing on going from small–medium chains (C4:0 esters 0.28% wt, C6:0 esters 0.14% wt) to medium (C9:0 esters 0.07% wt, LA esters 0.15%) and long chains (C18:1 0.08% wt, C18:2 0.04% wt).

Therefore, the residual concentration of zinc in the products depends upon the solubility properties of the corresponding zinc(II) carboxylate in the ester produced.

**Figure 5:**
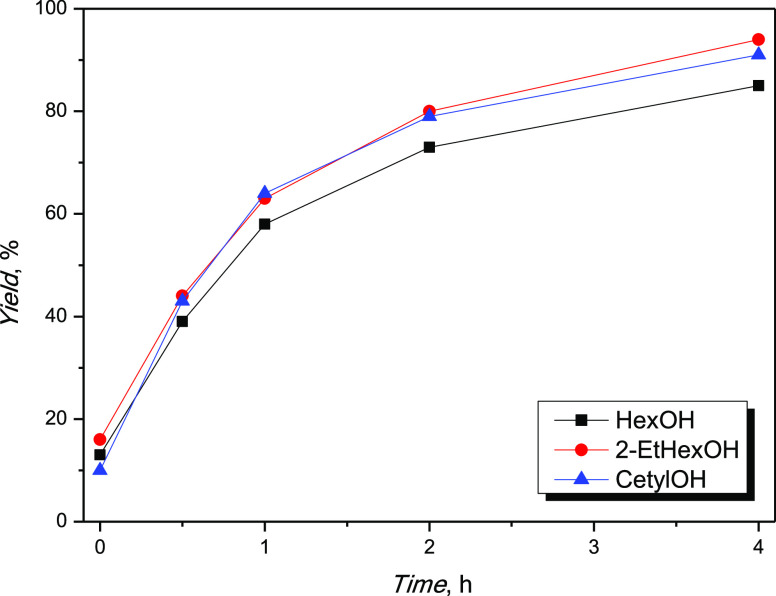
Alcohol screening.

For the screening of the alcohols, 1-hexanol (HexOH), 2-ethylhexanol (2-EtHexOH), and 1-hexadecanol (CetylOH) were used. The results are reported in Table 5. Figure 5 shows the yield trend as a function of time.

**Table 5:** Scope of Alcohols

		yield (%)^b^		
entry^a^	alcohol	2 h	4 h	[Zn] in product (ppm)^c^
1	HexOH	81	92	10.6
2	2-EtHexOH	80	94	1.4
3	CetylOH	79	90	191^d^

a Conditions: 170 °C, fatty acid to alcohol 1:1.2 mol/mol, and catalyst loading ZnO 1% mol.

b Thorough ^1^H NMR spectroscopy, relative error within 2%.

c ICP-MS analysis.

d Product filtered at 50 °C.

High yields are achieved within 4 h, and 2-ethylhexyl alcohol is converted faster. This circumstance is plausibly due to the greater volatility of hexyl alcohol and the high viscosity of the reaction mixture in the presence of cetyl alcohol, as it is solid at room temperature as well as the corresponding ester produced.

**Scheme 5:**
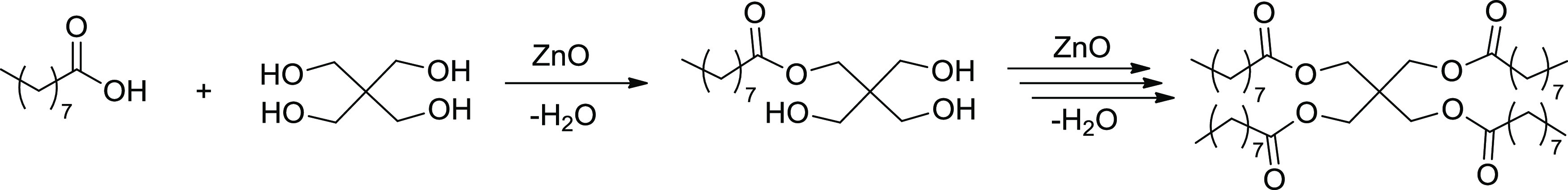
Pentaerythrityl Tetrapelargonate Synthesis

The efficiency of the catalytic system was also verified in the esterification of polyols, such as pentaerythritol and glycerol, whose esters find applications in different industrial sectors, such as in cosmetics and engine lubricants.^55^ The optimized molar ratio condition cannot be transferred to systems containing polyols to obtain complete conversion of the alcoholic groups. Therefore, an almost stoichiometric ratio was chosen as the best possible option, also to avoid subsequent harsh conditions to distill -off the excess of fatty acid. Scheme 5 shows the product formation pathway.

**Figure 6:**
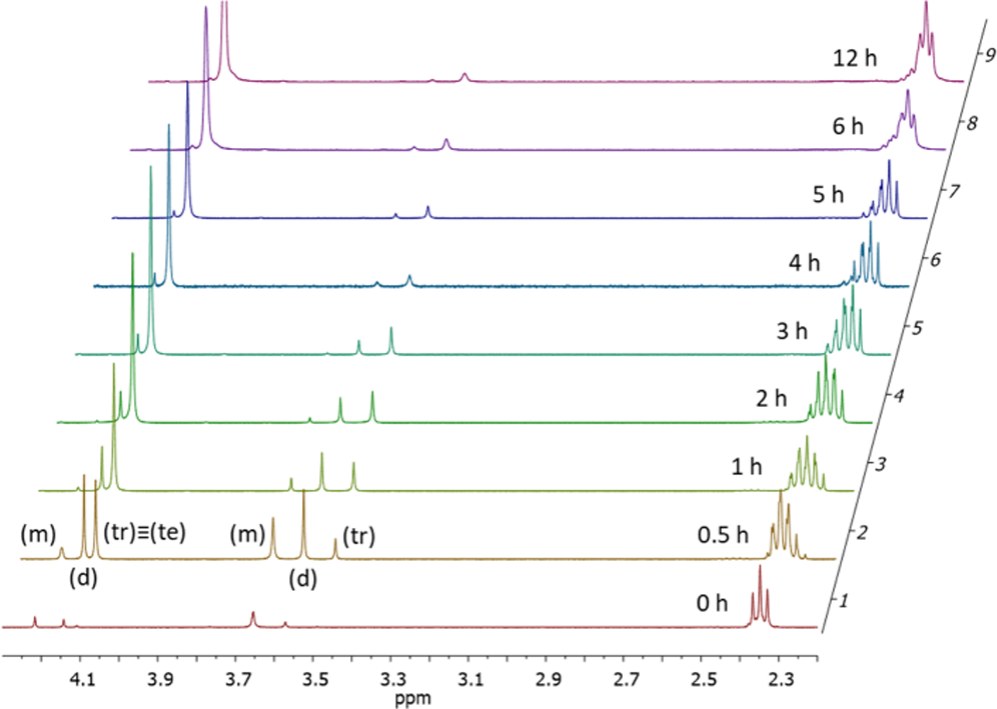
^1^H NMR monitoring of pentaerythrityl tetrapelargonate synthesis. Relevant signal attributions of pentaerythrityl pelargonate esters: (m) mono-CH_2_OC(O)R 4.19 ppm, CH_2_OH 3.65 ppm; (d) di-CH_2_OC(O)R 4.14 ppm, CH_2_OH 3.57 ppm; (tr) tri-CH_2_OC(O)R 4.11 ppm, CH_2_OH 3.49 ppm; and (te) tetra-CH_2_OC(O)R 4.11 ppm.

The reaction was carried out with pelargonic acid under almost stoichiometric conditions (acid ratio/alcohol = 4.1:1) and using ZnO at 1% mol with respect to the acid. Relevant portions of the proton NMR spectra of the reaction mixture recorded over time are reported below (Figure 6).

**Figure 7:**
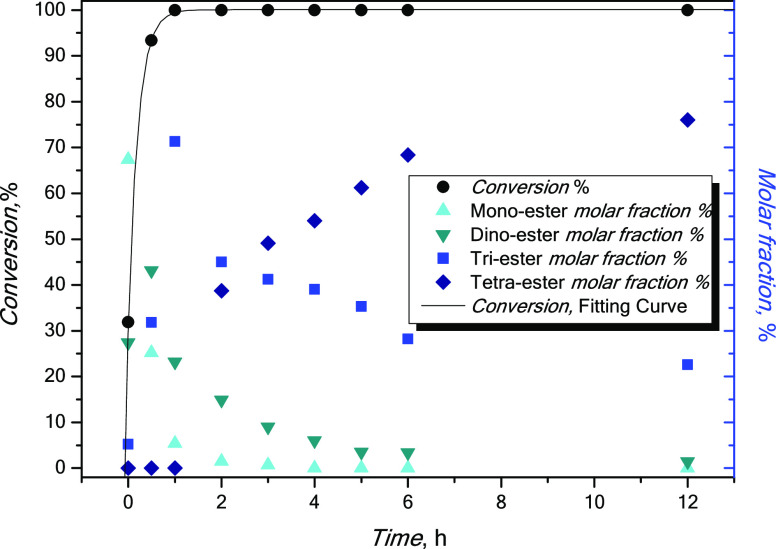
Pelargonic acid conversion and pentaerythrityl mono-, di-, tri-, and tetrapelargonate molar fraction over time.

From Figure 6, it is possible to clearly distinguish the alcoholic signals of pentaerythritol and of the various substituted species. Table S1 and Figure 7 show the product distribution of pentaerythrityl mono- and polysubstituted esters as a function of time. As in the case of monoalcohols, the spent catalyst can be easily recovered from the reaction mixture upon filtration at room temperature, with a residual Zn concentration in the product of 6.6 ppm.

**Scheme 6:**
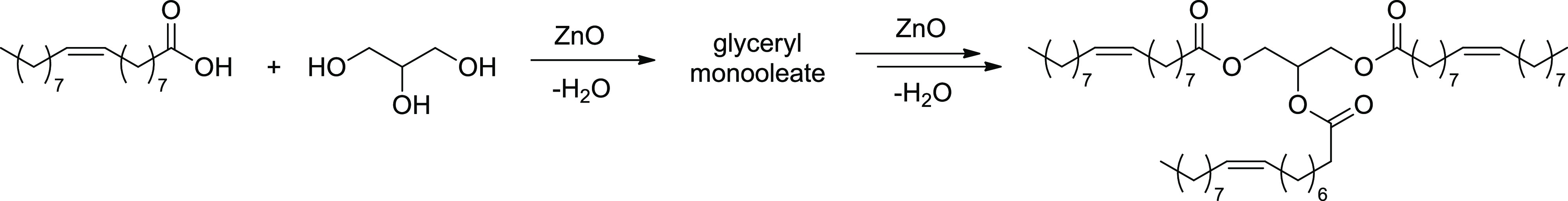
Glycerol Trioleate Synthesis

The reaction with glycerol was carried out with oleic acid in a ratio of 3.1:1 acid/alcohol and 1% ZnO with respect to the acid (Scheme 6). A high yield (>99%) was obtained in 4 h at 170 °C. Figure S5 shows a section of the ^1^H NMR spectrum recorded at 4 h.

The zinc content found in the produced esters is perfectly compatible with many applications in which medium–long-chain fatty acid esters are employed. Indeed, in food packaging, the specific migration limit (applicable to printed articles, expressed as mg of the substance per kg of food) of zinc is 5 ppm;^56^ in skin care cosmetics,^57^ the zinc(II) compounds are widely used as active or supportive ingredients; and in lubricating oils,^58^ the zinc carboxylates are used to improve the products.

Furthermore, the zinc content generally found in commercial olive oils is 20–70 ppm,^59^ a range higher than the residual concentration found in glyceryl trioleate (2.5 ppm).

### Catalyst Stability and Recycling

The simple recoverability of the spent catalyst and the low residual concentration of zinc(II) in the filtered product lead us to investigate the recyclability of the active species. A set of consecutive pelargonic acid esterification reactions with 2-ethylhexyl alcohol were performed in the optimized conditions. After each run, the catalyst was recovered by filtration and completely reused for the next run without any reactivation procedure. For the sole purpose of weighing the recovered catalyst, the latter one was washed with organic solvents (ethyl acetate and *n*-hexane) and then dried under reduced pressure.

**Figure 8:**
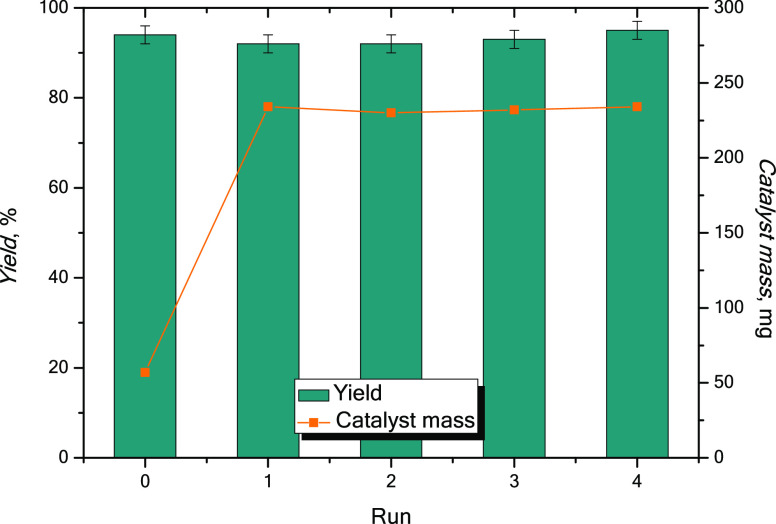
Recycling of the catalyst: the yield of the product and the mass of the catalyst over recycle runs. Conditions: 170 °C, 4 h, and fatty acid to alcohol 1:1.2 mol/mol. For run 0, catalyst loading was ZnO 1% mol, while other runs were done with the recovered spent catalyst.

Figure 8 shows the yields obtained at 4 h and the mass of catalyst recovered after each run.

The catalyst is plainly recyclable without any significant loss of activity (94, 92, 92, 93, and 95%, respectively). This circumstance is favored by its solubility in the reaction conditions, a prerogative that consents the catalysis to be effectively homogeneous.

The mass difference between the fresh catalyst (57 mg, 0.7 mmol) and the spent catalyst (234 mg) after run 0 highlights an increase in the mass due to the formation of the zinc carboxylate.

After run 0, the recycled mass is perfectly preserved in accordance with the ICP data relating to the residual concentration of the Zn(II) ion in the product.

**Figure 9:**
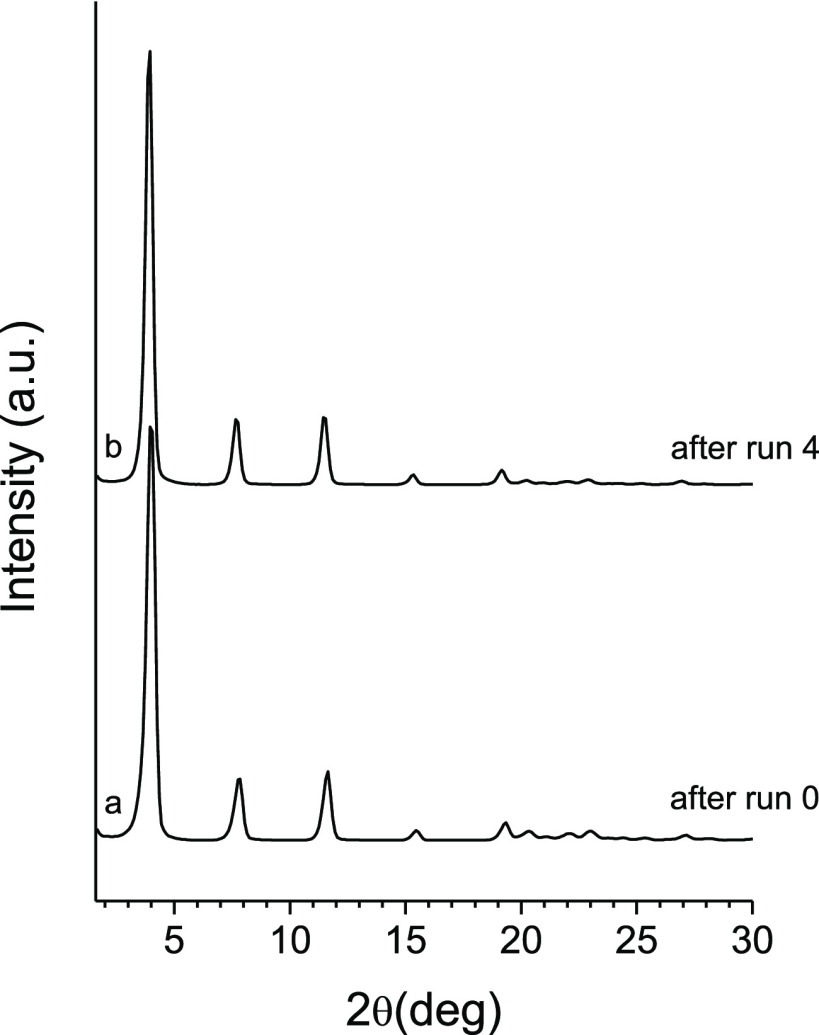
XRD analysis of the spent catalyst (a) after run 0 and (b) after run 4.

Finally, it is worth pointing out that by comparing the X-ray diffraction pattern of the spent catalyst recovered after run 0 with that of the solid recovered after four reaction cycles, it is possible to conclude that the catalyst remains unaltered after several runs (Figure 9).

### Reaction Pathway

**Scheme 7:**
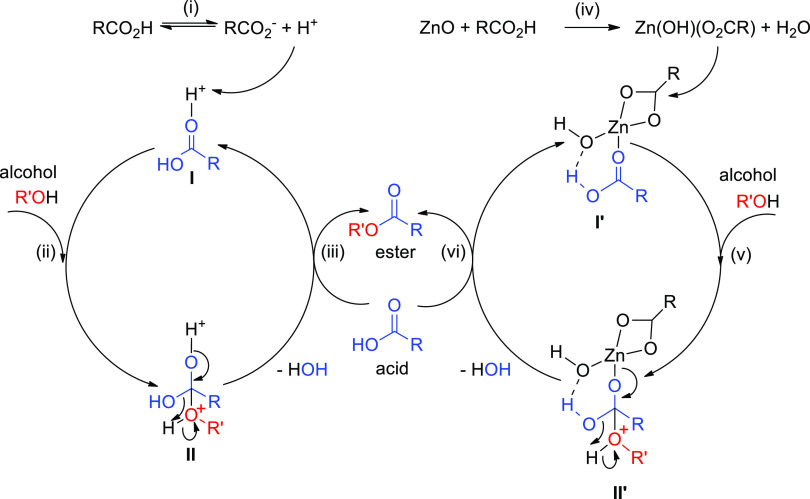
Proposed Pathway of the Esterification Reaction

The overall reaction pathway proposed in Scheme 7 is a revisitation of literature reports.^60,61^ As anticipated above, the reaction is initially supported by the Brønsted acidity of the system, as the H^+^ ions generated by equilibrium (i) promote the reaction according to the Fischer mechanism described by the left circle (steps ii–iii through intermediates **I**–**II**). As the reaction proceeds, the Lewis contribution (right circle) becomes relevant by zinc carboxylate species obtained through exchange reactions (as **I′** from step iv): the acid carbonyl is activated against the nucleophilic attack of the alcohol (v) through coordination to the metal ion (**I′**), and rearrangement of intermediate **II′** forms the ester (vi).

## Conclusions

This study demonstrates that simple zinc(II) salts allow the convenient esterification of fatty acids with medium-/long-chain alcohols and polyols. An in-depth investigation has disclosed that Zn(II) salts with basic Brønsted counterions (oxide, acetate, and carbonate) are plainly soluble in the hot reaction mixture, where they behave as homogeneous catalysts. Upon cooling, they precipitate as carboxylates, and, in this form, they are fully recoverable and recyclable many times without loss of activity. This beneficial prerogative couples the efficiency of the homogeneous catalysis with the advantage of the heterogeneous catalysis. Moreover, the absence of solvents or other auxiliary substances, the *E*-factor equal to zero, and the vegetable origin of the feedstock demonstrate the true sustainability of the overall manufacturing and set the basis for the scale-up of an innovative process to obtain commodities from renewable sources.

## Supporting Information Available

Relevant ^1^H NMR, FT-IR, and XRD spectra (PDF)

## Abbreviations

FA: fatty acid
NMR: nuclear magnetic resonance
ICP-MS: inductively coupled plasma mass spectrometry
MR: molar ratio
PA: pelargonic acid
LA: levulinic acid
HexOH: 1-hexanol
2-EtHexOH: 2-ethyl-1-hexanol
CethylOH: 1-hexadecanol
