# Supplementary material for: Homogeneous Catalysis and Heterogeneous Recycling: A Simple Zn(II) Catalyst for Green Fatty Acid Esterification
Source: ACS Sustain Chem Eng. 2021 Apr 22;9(17):6001–11. doi: 10.1021/acssuschemeng.1c01140 (PMC8297397; doi:10.1021/acssuschemeng.1c01140)
Supplement: Supplementary file 1 — sc1c01140_si_001.pdf [file sc1c01140_si_001.pdf]

Homogeneous catalysis and heterogeneous recycling: simple Zn(II) catalyst for green fatty acids esterification

*Massimo Melchiorre,[a] Maria Elena Cucciolito,[b, c] Martino Di Serio,[b, c] Francesco Ruffo,[b, c] Oreste Tarallo,[b] Marco Trifuoggi[b] and Roberto Esposito[b, c, *]*

[a] ISUSCHEM S.r.l., Piazza Carità 32, 80134, Napoli, Italy.

[b] Dipartimento di Scienze Chimiche, Università di Napoli Federico II, Via Cintia 21, 80126, Napoli, Italy.

[c] Consorzio Interuniversitario di Reattività Chimica e Catalisi, Via Celso Ulpiani 27, 70126, Bari, Italy.

*roberto.esposito@unina.it

Number of pages: 5

Number of figures: 5

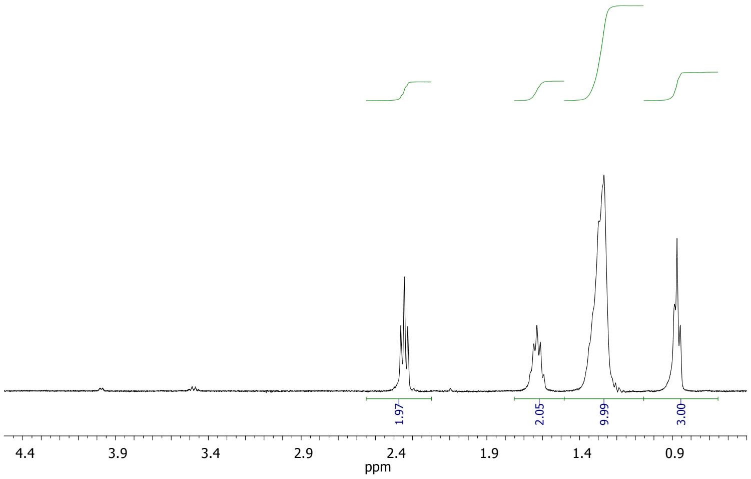

Figure S1: Relevant ^{1}H-NMR spectrum portion of spent catalyst (Table 2 Entry 2).

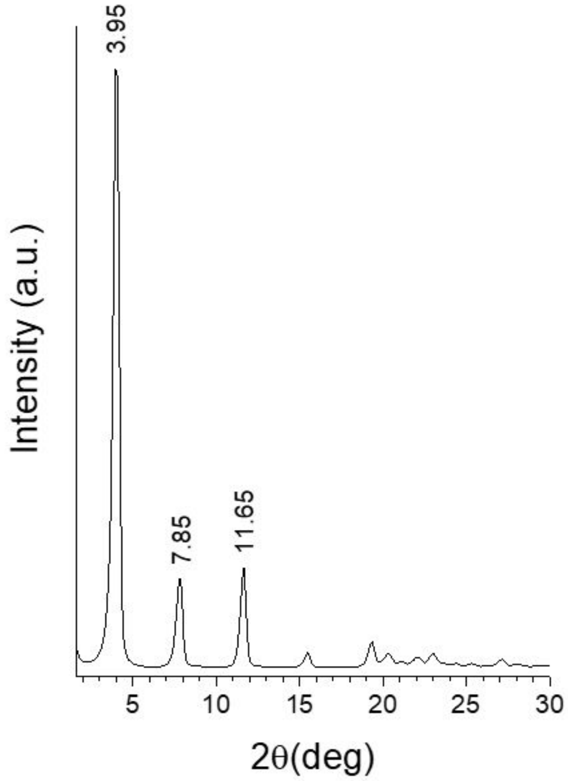

Figure S2: X-ray powder diffraction patterns of spent catalyst (Table 2 Entry 2).

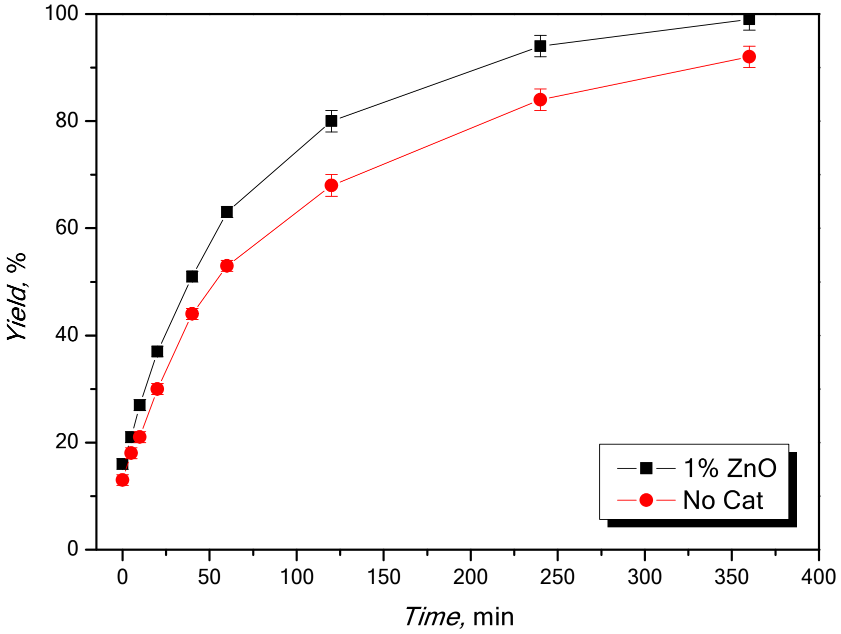

Figure S3. Yield over time plot of Entry 1 and Entry 3 of Table 2.

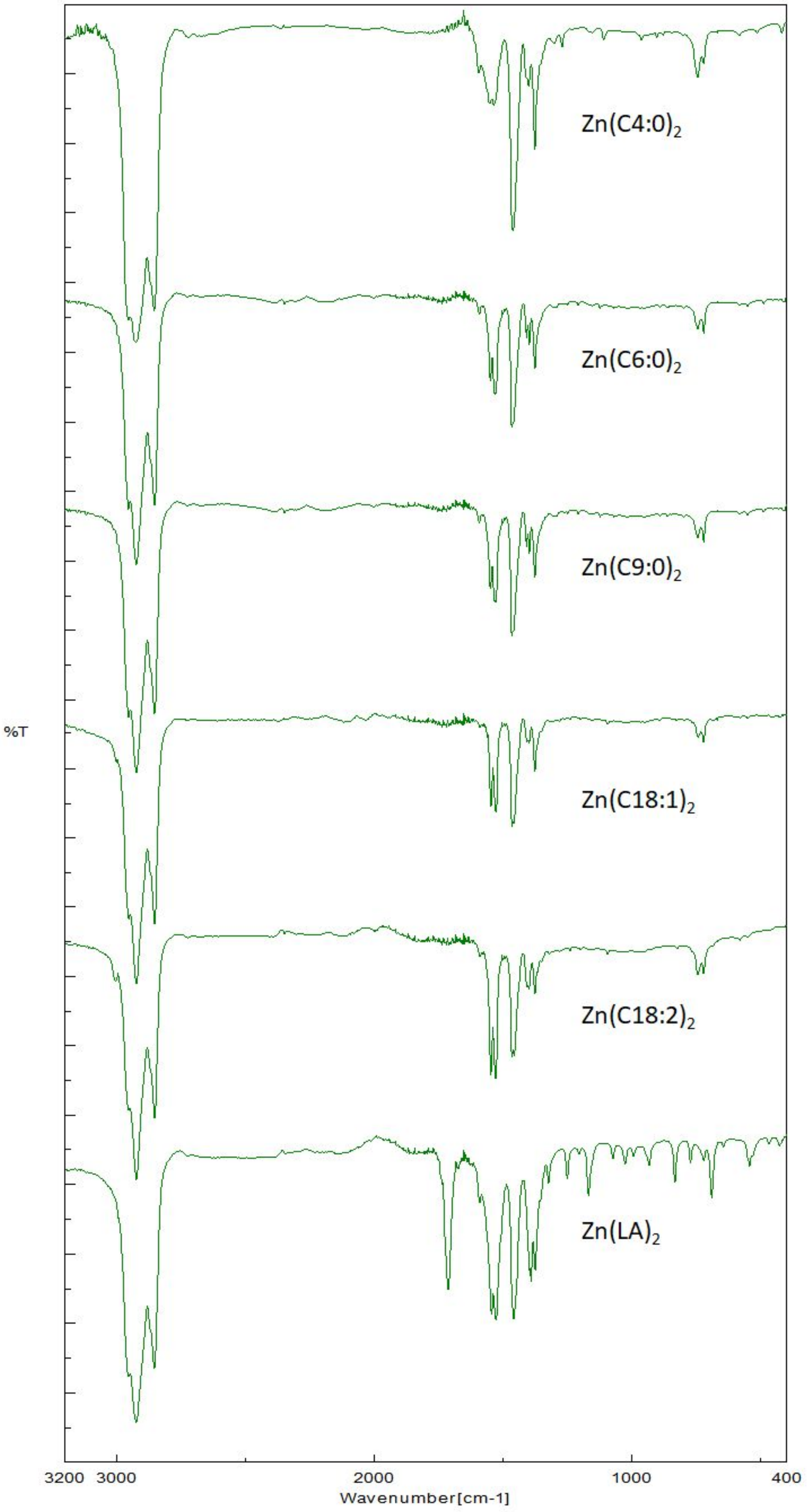

Figure S4: Relevant portion of FT-IR spectra of spent catalysts characterized as zinc carboxylates.

Table S1. Molar fraction monitoring of pentaerythrityl mono- di- tri- tetra- pelargonate.

Time (h)[a]	X mono-ester	X di-ester	X tri-ester	X tetra-ester	Conversion (%)[b]
0	68	27	5	0	32
0.5	25	43	32	0	93
1	6	23	71	0	100
2	1	15	45	39	100
3	<1	9	41	49	100
4	<1	6	39	54	100
5	0	3	35	62	100
6	0	3	28	69	100
12[c]	0	1	23	76	100

[a] Conditions:170°C, fatty acid to alcohol 4.1:1 mol/mol, catalyst loading ZnO 1% mol respect to the acid; [b] thorough ^{1}H NMR spectroscopy, relative error within 2%; [c] the filtered product contains 6.6 ppm of zinc (ICP-MS analysis).

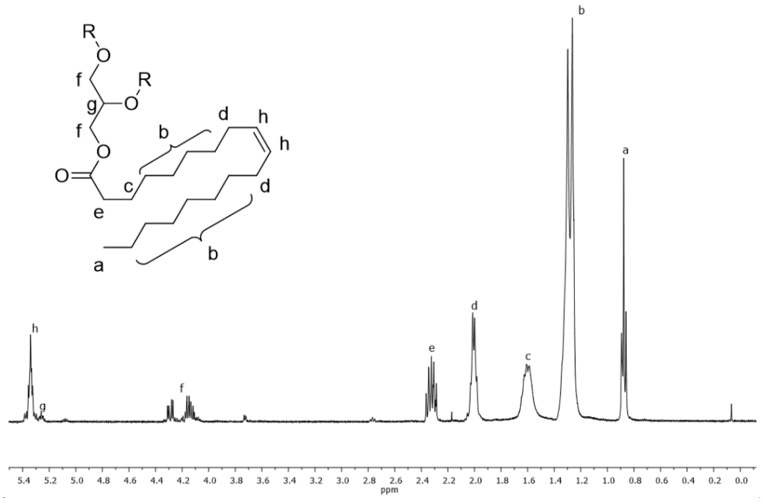

Figure S4: Relevant ^{1}H-NMR spectrum portion of glyceryl trioleate obtained as described in scope paragraph.
